# Droplet-Based Microfluidic Thermal Management Methods for High Performance Electronic Devices

**DOI:** 10.3390/mi10020089

**Published:** 2019-01-25

**Authors:** Zhibin Yan, Mingliang Jin, Zhengguang Li, Guofu Zhou, Lingling Shui

**Affiliations:** 1 Guangdong Provincial Key Laboratory of Optical Information Materials and Technology & Institute of Electronic Paper Displays, South China Academy of Advanced Optoelectronics, South China Normal University, Guangzhou 510006, China; jinml@scnu.edu.cn (M.J.); 2018023161@m.scnu.edu.cn (Z.L.); guofu.zhou@m.scnu.edu.cn (G.Z.); 2 National Center for International Research on Green Optoelectronics, South China Normal University, Guangzhou 510006, China; 3 Defense Key Disciplines Lab of Novel Micro-Nano Devices and System Technology, Chongqing University, Chongqing 400044, China; 4 Shenzhen Guohua Optoelectronics Technology Co., Ltd., Shenzhen 518110, China; 5 Academy of Shenzhen Guohua Optoelectronics, Shenzhen 518110, China

**Keywords:** microfluidics, droplet, electrowetting, integrated/embedded thermal management, hot spots, 3D IC packaging

## Abstract

Advanced thermal management methods have been the key issues for the rapid development of the electronic industry following Moore’s law. Droplet-based microfluidic cooling technologies are considered as promising solutions to conquer the major challenges of high heat flux removal and nonuniform temperature distribution in confined spaces for high performance electronic devices. In this paper, we review the state-of-the-art droplet-based microfluidic cooling methods in the literature, including the basic theory of electrocapillarity, cooling applications of continuous electrowetting (CEW), electrowetting (EW) and electrowetting-on-dielectric (EWOD), and jumping droplet microfluidic liquid handling methods. The droplet-based microfluidic cooling methods have shown an attractive capability of microscale liquid manipulation and a relatively high heat flux removal for hot spots. Recommendations are made for further research to develop advanced liquid coolant materials and the optimization of system operation parameters.

## 1. Introduction

**Figure 1:**
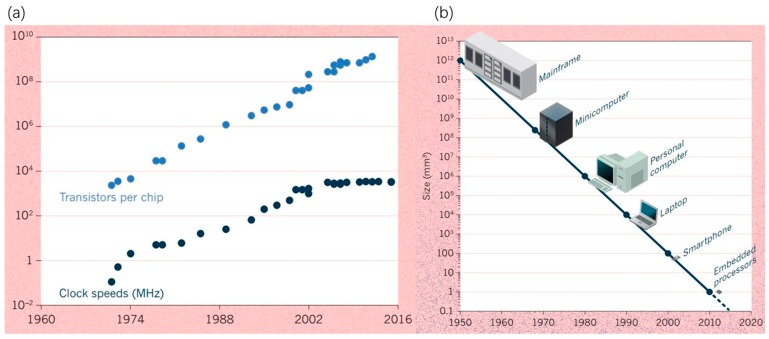
Trends of (**a**) the number of transistors per microprocessor chip, clock speeds, and (**b**) the size of machines every 10 years. Reproduced with permission from [3], published by Nature, 2016.

In 1965, Gordon Moore, the co-founder of Fairchild Semiconductor and chief executive officer (CEO) of Intel estimated that the number of transistors and other electronic components per integrated circuit was doubling every year [1]. Later on, he revised his forecast rate to doubling every two years in 1975 [2]. It gradually became the well-known “Moore’s law” in the electronics industry. In consideration of increasing the performance of transistors, David House, an Intel colleague, concluded that the performance of an integrated circuit and chip would double every 18 months. Moore’s famous law has powered the information and communication technology revolution since the 1960s. However, it seems to have met its demise [3]. Although the number of transistors per chip has been following Moore’s law, the maximum clock rate has not budged since 2004, as shown in Figure 1a. Heat death has been the major obstacle, while the feature size of electronic devices continues to reduce (shown in Figure 1b). Microelectronic chips have become too hot due to the increased transistor density when silicon element sizes become smaller and smaller. If waste heat cannot effectively dissipate from chips, the whole device will burn down in seconds. Thus, advanced thermal management methods are in high demand in order to extend Moore’s law progression in the electronics industry. Currently, three major cooling challenges are the effective removal of high heat flux, non-uniform power dissipation (e.g., hot spots), and confined space constraints (e.g., 3D stacked packaging). As shown in Table 1, vigorous research efforts have been made on various thermal management methods with a high cooling capacity in past decades, including microchannel cooling [4], spray cooling [5,6] and jet impingement cooling [7], and surface wettability modification [8]. Ultra-high removal heat flux up to 2000 W/cm^2^ has been achieved using impingement jets with liquid metal [9]. Three-dimensional integrated circuit (3D IC) packaging is recognized as a promising solution to sustain Moore’s law progression. The three-dimensional stacking of multiple cores and units vertically as a single chip can further increase overall clocking speed without reducing the microelectronic element size. Meanwhile, the 3D-stacked configurations lead to significant waste heat accumulation between different stacks, and the formation of hot spots with extremely high local heat flux. Non-uniform temperature distribution and hot spots in devices have become major challenges for 3D IC packaging devices [10].

As illustrated in Table 1, several cooling techniques, such as pool boiling, spray cooling, and jet impingement, can dissipate waste heat more effectively than traditional air-based cooling techniques. However, most of these techniques require a relatively large space or have complex system architectures. They are inherently difficult to be integrated into circuits and chips, especially to cool the hot spots in the extremely confined space inside the 3D stacked packages. Therefore, effective integrated/embedded thermal management methods have gained increasing interest to overcome the physical constraints in electronic devices with shrinking sizes, which cannot be tackled by conventional cooling technologies [10]. Microfluidic cooling systems based on semiconductor fabrication technologies enable miniaturization of the system size and easy integration into electronic devices, which offer promising solutions to on-chip or in-chip cooling for 3D IC packaging. The intra/inter chip enhanced cooling thermal packaging program was launched by the USA Defence Advanced Research Agency (DARPA) in 2012. The program proposes to employ microfluidic technology to directly cool the waste heat dissipated in the chip and package based on the unique advantage of microfluidics, such as their compact size (10–1000 µm) and precise manipulation of a small amount of liquids [10].

Currently, microfluidic thermal management methods normally consist of two types: a continuous liquid phase and a discrete liquid phase. Microchannel cooling and microjet impingement cooling continuously supply liquid convective flow or liquid jets into the hot zones, respectively, and waste heat is dissipated out of the chip as liquid flows out after the heat transfer process. Droplet-based microfluidic cooling techniques utilize discrete droplets [19,20] and liquid plugs [21,22] to remove waste heat from the hot zones. Since Tuckerman and Pease [23] first proposed using microchannel heat sinks to cool high-power-density electronic devices in 1981, the heat removal capacity of advanced thermal management systems has significantly enhanced from 100 W/cm^2^ by forced-air convection up to 790 W/cm^2^. The compact size of microchannels enables the feasibility of the direct integration of cooling units with electronic packages, which is believed to be a potential solution to the rapid increment of waste heat dissipation in advanced integrated circuit development. However, coolants require external pumps generating large pressure driving force in the microchannels. In order to further enhance the heat removal capacity of microchannel heat sinks, the flow rates of coolants need to be increased, which inevitably results in increased power consumption and an increased pump size. The same challenge is faced by microjet impingement cooling and liquid slug/plug cooling techniques. Discrete droplet cooling techniques driven by surface-tension modulation provide an energy-friendly option for an integrated on-chip cooling system. Without the requirement of powerful liquid pump and complex tubing connections, a high coolant flow rate can be achieved by periodic modulation of the surface tensions of the front and back menisci of each discrete microdroplet according to external applied fields [19]. Different methods of surface-tension modulation for droplet actuation have been proposed, such as thermal, electrostatic, and chemical methods. To be more specific, electrostatic surface-tension modulation refers to the electrocapillary principle in this work, which has been the most flexible and most functional for droplet driving mechanisms of digital microfluidic (DMF) lab-on-a-chip devices [24]. The electrocapillary-based methods can be the most promising option for thermal management due to fast response times, low power consumption, and low heat generation, compared to thermal and chemical methods.

In this work, we review the fundamental theory of droplet-based microfluidic thermal management methods and recent developments that are promising for integrated/embedded thermal management systems of high-performance electronic devices. Emphasis is placed on the electrocapillary-principle-related methods and other droplet-based methods based on surface-tension modulation. Technologies employing microdroplets in spray cooling are generally not covered, as they require a relatively high-power conventional mechanical actuator for droplet generation and transport. Relevant information is recommended to refer to previous reviews [5,25]. Section 2 focuses on the basic theory of electrocapillary and its categorization. Section 3 presents the developments of electrocapillary-based active thermal management studies. Section 4 describes recent works about passive cooling techniques based on the jumping-droplets phenomenon. Concluding remarks and an outlook for future development are provided in Section 5.

## 2. Electrocapillarity

**Figure 2:**
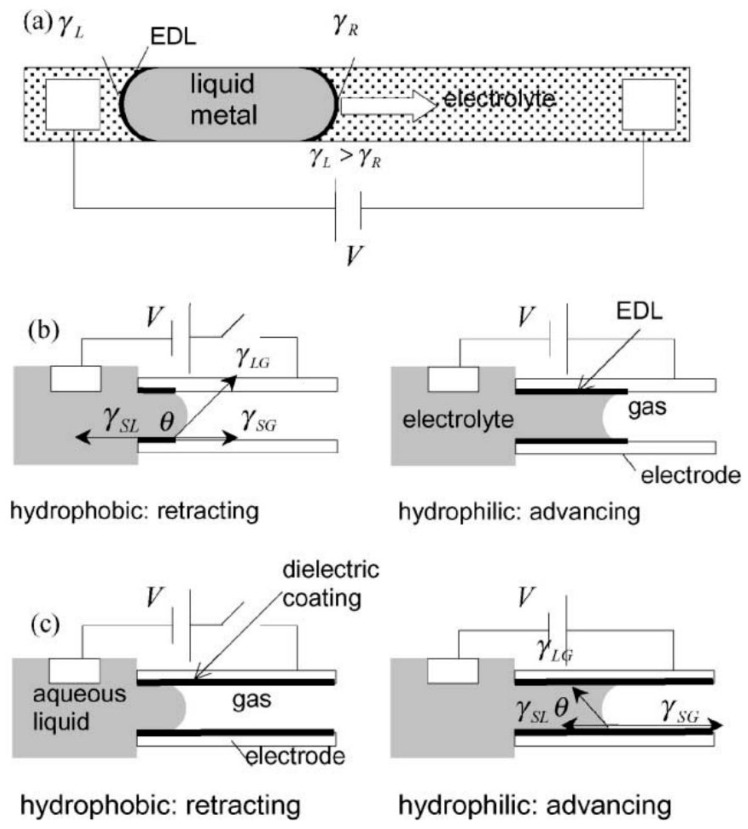
Schematics of electrocapillary phenomena: (**a**) continuous electrowetting (CEW); (**b**) electrowetting (EW); (**c**) electrowetting-on-dielectric (EWOD). Reproduced with permission from [27]; published by ELSEVIER, 2002.

In 1875, Gabriel Lippmann, a Nobel laureate in Physics 1908, first discovered electrocapillarity, a process by which the interfacial tension of a liquid metal (e.g., mercury) suspended in an inert electrolyte can be changed under an electrical potential applied to a liquid metal [26]. As shown in Figure 2a, an electrical double layer (EDL) is formed at the metal–electrolyte solution interface, and the charge density in the EDL is changed as a function of the applied electrical potential. When the capacitive energy at the EDL is reduced with the variation of the charge density, the surface area of the liquid metal is increased and the effective interfacial tension at the metal–electrolyte solution interface is changed accordingly. Consequently, a motion of liquid metal occurs caused by the pressure difference, which results from the difference between interfacial tensions at the two menisci under the applied electrical potential.

Depending on the configuration of the interfacial system, the electrocapillary phenomena can be categorized into three types: continuous electrowetting (CEW), electrowetting (EW), and electrowetting on dielectric (EWOD), as illustrated in Figure 2 [27]. The work of Lippmann using liquid metal in aqueous electrolytes belongs to the CEW category (Figure 2a). For CEW, the liquid metal does not directly contact the electrode and is actuated by the changes of the liquid metal–electrolyte surface tension at the liquid–liquid interface. The terms “electrowetting” and “electrowetting on dielectric” are often used interchangeably in some literatures, but they are different. “Electrowetting on conductor” was also used in [28] and can be treated as electrowetting in this review. Here, we differentiate the two terms following the description proposed by Lee et al. [27]. For EW and EWOD, the liquid motion is caused by the changes of liquid–solid interfacial tensions at the liquid–solid–gas interfaces as shown in Figure 2b,c, respectively. In EW, liquid–solid interfacial tension is changed at the EDL formed at the interface between electrode and electrolyte, while the change of liquid–solid interfacial tension in EWOD occurs in a hydrophobic dielectric layer instead of the EDL in EW [27]. The relationship between the interfacial tension and the applied electrical potential can be expressed as Lippmann’s equation [26]:

(1) $$\gamma =\gamma_{0}-\frac{1}{2}cV^{2}$$

where $\gamma_{0}$ is the interfacial tension without the electrical field applied across the interface, and *c* is the capacitance per unit area of EDL for CEW and EW, or the dielectric layer for EWOD. It should be noted that the interfacial tension (γ) is the surface tension at the liquid–liquid interface between the liquid metal and the electrolyte for CEW (shown in Figure 2a), while it becomes the surface tension ($\gamma_{SL}$) at the solid–liquid interface for EW and EWOD. Since the contact angle (θ) can be derived in terms of surface tension by Young’s equation as

(2) $$\gamma_{SG}=\gamma_{SL}-\gamma_{LG}cos\theta ,$$

Lippmann’s equation can be expressed with contact angle as

(3) $$cos\theta =cos\theta_{0}+\frac{1}{2\gamma_{LG}}cV^{2},$$

where $\gamma_{SG}$ and $\gamma_{LG}$ are the surface tensions at the solid–gas and liquid–gas interfaces, respectively, $\theta_{0}$ is the contact angle without an electrical field applied across the interface. Equation (3) is called the Young–Lippmann equation, in which the contact angle is clearly a function of the applied electrical potential. Thus, the surface wettability of a liquid droplet can be regulated by applying the electrical field across the interface. The transport of discrete liquid droplets can be readily manipulated by controlling the applied electric field according to application requirements, including flow rate, transport speed, and route.

## 3. Active Droplet-Based Microfluidic Methods: Electrocapillary-Based

This section presents research on active droplet-based microfluidic cooling methods based on electrocapillary phenomena. It is divided by two subsections: CEW-based and EW- and EWOD-based applications.

### 3.1. Continuous Eletrowetting (CEW)

In 2000, Lee and Kim [29] demonstrated the first MEMS device, which actuated liquid metal microdroplets based on the CEW principle, and they showed smooth and wear-free droplet motion at a speed of about 40 mm/s with an applied electrical potential of 2.8 V. Their works confirm the feasibility of effective handling microscale liquid droplets with low power consumption (10–100 µW). Thereafter, active thermal management based on the CEW principle was first proposed by Mohseni [30] in 2005. Discrete droplets of liquid metal/alloy are actuated and flowing along the electrode patterns due to electrical modulation of the surface tension, which is achieved by switching on/off the electrodes with a specific sequence as illustrated in Figures 2 and 3 in Ref. [30]. This successfully demonstrates the potential of CEW as an effective cooling technique for high-power-density electronic packages and the removal of hot spots in devices. In an analogous method of digital microfluidics, Mohseni and Baird [20] named this cooling technique digitized heat transfer (DHT). In DHT, thermal energy (i.e., waste heat) is transported out of the device in a discrete manner, as individual droplets are actuated by electrodes in sequence. It is fundamentally different from the conventional cooling techniques using conduction by conductive solid and convection by continuous liquid flow.

**Figure 3:**
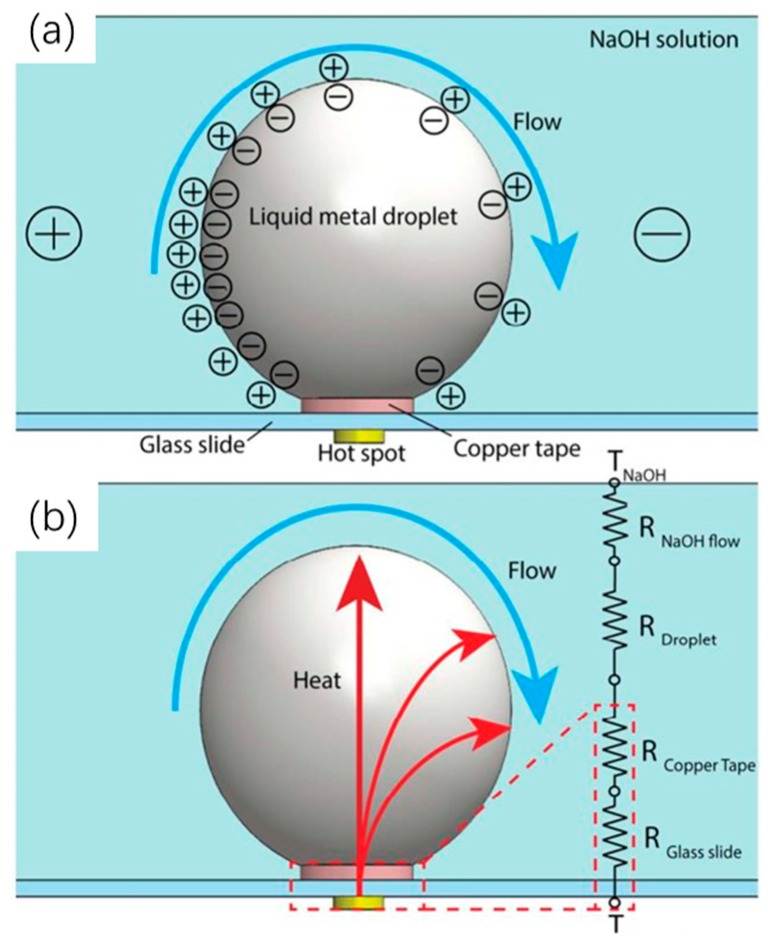
Schematic of mechanism of electrical actuated liquid metal droplet on a hot spot based on the Marangoni effect. (**a**) The generation of harmonic Marangoni flow and surface charge distribution of liquid metal droplet when an electric potential is applied between the electrodes. is enabled by continuous electrowetting effect at the surface of the liquid metal droplet, upon the application of a square wave DC signal. (**b**) Schematic and equivalent thermal circuit of heat dissipation through liquid metal droplet, where T is temperature and R is thermal resistance. Reproduced with permission from [32]; published by ACS Publications, 2016.

Due to its fluidic property at room temperature and high thermal conductivity, mercury has been frequently used to demonstrate CEW-based electrical control of discrete droplet motion and DHT for compact thermal management applications. However, mercury is a toxic heavy metal which is not appropriate for general use in cooling applications. The liquid alloys have low melting points below or near room temperature and a good thermal property, as shown in Table 2 [31]. Liquid alloys can be a good substitute to replace mercury in CEW-based thermal management systems. Thus, CEW-based cooling techniques can be further developed without the environmental and health risks. Zhu et al. [32] recently used a Galinstan (Ga) droplet sitting on a hot spot as a CEW-based cooling system, and the Galinstan droplet actuated by a square wave signal was acting as a “soft pump” in their designed cooling system (shown in Figure 3). Due to the square wave signal, a surface tension gradient was formed across the droplet, and a Marangoni flow was induced surrounding the droplet. As a result, the flow rate of the coolant around the Galinstan droplet can be increased by the Marangoni flow. Meanwhile, the Galinstan droplet can facilitate the waste heat conducted from the hot spot to the coolant due to its excellent thermal conductivity. The over single-phase heat transfer performance is consequently improved.

### 3.2. Electrowetting (EW) and Electrowetting on Dielectric (EWOD)

After Lippmann discovered the electrocapillarity (i.e., CEW), numerous works have been performed on liquid metal (e.g., mercury and liquid metal alloy) suspended in different aqueous electrolytes. Further development of CEW in practical applications has been impeded by its intrinsic disadvantage of the electrolytic decomposition of water. Water would easily decompose under an applied electrical potential of more than a few hundred millivolts [34]. Until the 1980s, noticeable attention was paid to Lippmann’s discovery when the term “electrowetting” (EW) was coined and when EW was used for designing display devices by Beni and Hackwood from Bell Laboratories [35,36,37]. In their studies, they pointed out that electrocapillarity (CEW) and EW were distinct, and defined EW as the change in solid–liquid contact angle due to applied electrical potential difference across the solid–liquid (i.e., electrode–electrolyte) interface. The wettability of a certain electrolyte on an electrode in EW is changed by varying the electric energy across the EDL [20,27]. However, electrolysis still exists in EW since the liquid electrolytes directly contact with the conducting electrode. In 1993, Berge [38] proposed the implementation of a thin dielectric coating layer on metallic electrodes, which can prevent the conductive liquid (e.g., electrolyte solution) from directly contacting the electrode. As a result, the problem of water electrolysis was successfully solved. This technique is known as EWOD, which profoundly expands the applications of electrocapillarity for handling liquid on a microscale. EWOD can deal with almost any aqueous liquid through modulation of electric energy across the dielectric coating layer. As a result, most of the recent studies and applications about droplet-based methods are performed on dielectric. A detailed review of the basis of EWOD can be found in [34,36].

Chakrabarty’s research group from Duke University [19] are the pioneers for employing the EWOD technique in the thermal management of high-power-energy density electronic devices as illustrated in Figures 1–3 in Ref. [39]. They first fabricated an open digital microfluidic chip using a standard printed circuit board (PCB) process, and brush-coated a thin layer of Teflon^®^ AF on the PCB board to ensure surface hydrophobicity. Conducting liquid droplets (1M KCl, 6 µL) were placed on the Teflon^®^ AF coating layer and electrostatically actuated to transport across the hot spot (65 °C) at a switching frequency of 32 Hz, and the hot spot was cooled by 20 °C within 0.24 s [40]. Silicon oil was added into the KCl microdroplets to prevent water evaporation and kept a constant droplet volume. They also demonstrated that high volume flow rates of discrete microdroplets could be achieved in excess of 10 mL/min [19]. Using this experimental setup, they performed parametric investigations on the effects of droplet flow rate, droplet aspect ratio [40], switching frequency, hot-spot heat flux density, and droplet volume [41]. Based on the EWOD principle, two major hot-spot cooling schemes were proposed by Paik et al. [39], including flow-through adaptive cooling (shown in Figure 2 in Ref. [39]) and programmable thermal switch (shown in Figure 3 in Ref. [39]). A flow-through adaptive cooling scheme utilizes a continuous droplet train transporting from a coolant reservoir to hot spots in two dimensions on hot electronic device surfaces. The waste heat generated by the hot spots would be carried away by the coolant droplets, and the coolant droplets are cooled back to original states after heat transfer with the coolant reservoir. As such, this closed-loop adaptive cooling system can be used to eliminate thermal nonuniformity caused by hot spots. A programmable thermal switching scheme takes advantage of EWOD from a different perspective, which electrostatically manipulates the wettability of liquid metal droplets (e.g., mercury) to selectively switch an overheated zone from a high thermal-conductivity state to a higher state. The liquid metal droplets were uniformly dispensed into an array of cooling cells on the electronic device surface. The droplets work as an active thermal conductor or interface between the device surface and the heat sink. The switching state can be changed according to the overheating degree of a specific zone on the device surface, which enables precise hot-spot cooling on demand. Recently, Alavi et al. [42] also used EWOD-actuated mercury droplets as a thermal switch for hot-spot cooling, and performed numerical simulations to further study the effects of different system parameters, such as surrounding fluid, contact radius of droplet with the top surface, and the occupied portion of the cell by the liquid droplet. In fact, variations of these parameters change the equivalent thermal conductivity of the liquid metal droplet.

After the EWOD-based cooling was successfully demonstrated by Pamula and Chakrabarty [19] in 2003, research in this field gained increasing attention. Baelmans et al. [43] performed an experimental study on the comparison of heat transfer performance between continuous liquid cooling (i.e., microchannels actuated by external pump) and discrete liquid cooling (i.e., droplets actuated by EWOD). Continuous microchannel cooling excelled in the reduction of thermal resistance down to 0.2 K/W at a pressure drop of about 0.5 bar. On the other hand, discrete droplet-based cooling provided a lower cooling rate but offered lower pumping power per mass flow rate and controllable cooling for specific overheated zones (i.e., hot spots) locally. Moreover, they applied an AC voltage signal in EWOD actuation of a sessile droplet on a microchannel heat sink surface as seen in Figure 4 in Ref. [44]. The microchannel sink can be periodically filled and emptied with the liquid droplet upon the application of an AC electric field. The cooling rate of this microchannel heat sink is enhanced with the help of liquid filling actuated by EWOD compared to cooling by conduction via bulk silicon substrate. Bahadur and Garimella [45] developed energy minimization-based modelling approach for EWOD-based hot-spot cooling applications, which can be employed as a preliminary criteria to design rough surfaces (e.g., microchannels shown in Figure 4 in Ref. [44]) with electrically tunable thermal resistance. Kumari and Garimella [46] performed further numerical investigation on the heat dissipation capacity of an EWOD cooling system based on their three-dimensional transient numerical modeling. With the same pumping power, the heat dissipation capacity of the EWOD system is comparable to microchannel cooling systems according to their simulation results.

**Figure 4:**
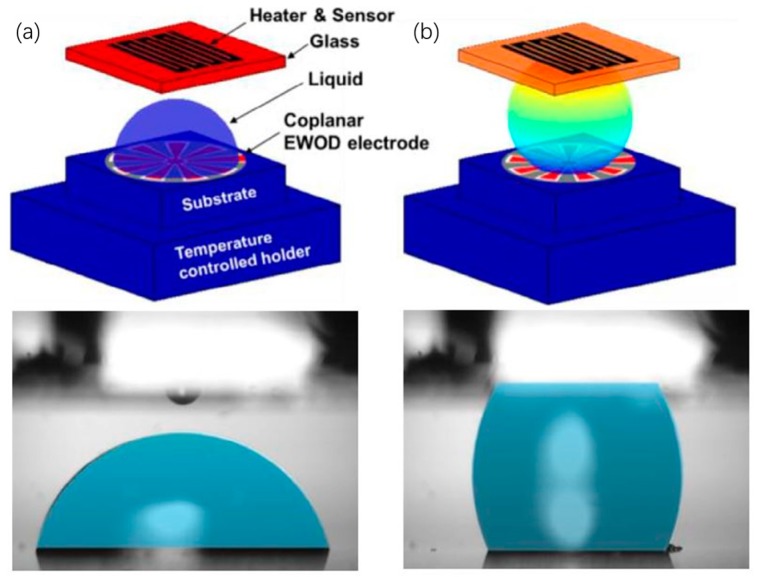
Thermal conductance switching based on the actuation of liquid droplets through EWOD principle with the thermal switch in the off (**a**) and on (**b**) states. Reproduced with permission from [48]; published by ELSEVIER, 2016.

At around the same period as Chakrabarty et al. in 2008, Kim’s research group from UCLA proposed and demonstrated the thermal switch cooling scheme using water and glycerin droplets actuated by coplanar EWOD chip as shown in Figure 4 [47]. After successful experimental demonstration, they conducted a numerical simulation to elucidate the mechanism of thermal performance measured in experiments, and pointed out that the thermocapillary flows plays a vital role in enhanced heat transfer of dielectric liquids [48]. Dielectric liquids with high Marangoni numbers could be a potential alternative to liquid metal for a thermal switch cooling scheme. Comparison among heat transfer performances of using different liquids in EWOD-based cooling system were performed by Moon et al. [49], including DI water and room temperature ionic liquids (e.g., [BMIM]Cl, [BMIM]Ntf2, and [CMIM]FeCl_4_). The overall heat transfer performance of the ionic liquids was not improved compared to that of DI water due to their low thermal conductivity and high viscosity. However, the ionic liquids demonstrated excellent thermal stability, as no evaporation occurred during the cooling process, while DI water showed vigorous evaporation under the same heat load. An attempt to increase the thermal conductivity of the ionic liquids was made by adding multiwall carbon nanotubes (MWCNTs) to the liquid droplets. Besides work on liquid coolants, efforts have also been made with respect to the fabrication of EWOD-based cooling devices. Cheng and Chen [18] designed and fabricated coplanar control electrodes, which enable every electrode unit with the capability of interchanging between activating and ground functions on demand. Moreover, carbon nanotubes (CNTs) grown on the cover electrode plate can significantly reduce the pressure drop for EWOD liquid transport due to the superhydrophobicity provided by CNTs.

**Figure 5:**
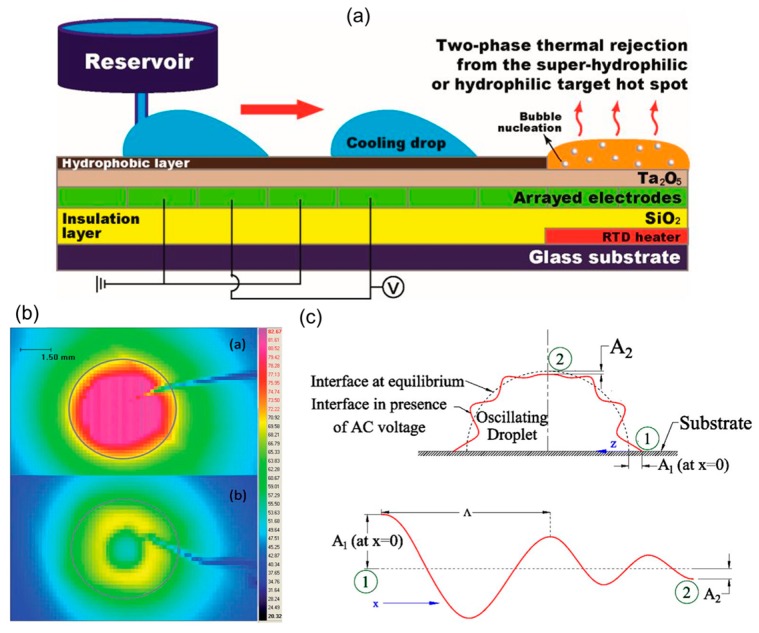
(**a**) Schematic of the single-sided digital microfluidic (SDMF) device and the working principle of effective liquid delivery and two-phase cooling; Reproduced with permission from [50]; published by MDPI, 2017. (**b**) schematic of oscillating droplet with the surface wave. Reproduced with permission from [55]; published by ELSEVIER, 2017; (**c**) temperature distributions at and around the hot spot with (bottom) and without (top) oscillating droplet. Reproduced with permission from [54]; published by The Royal Society of Chemistry, 2014.

Unlike the single liquid phase heat transfer of the EWOD-based flow-through cooling schemes mentioned aforementioned, Park et al. [50,51] utilized two-phase cooling in conjunction with EWOD microfluidic devices, which enabled the usage of a high latent heat during the phase change process of liquid droplets, as illustrated in Figure 5a. As a result, the cooling capacity of EWOD-based cooling system can be remarkably enhanced. Superhydrophilic modification of the hot-spot surface improved the adhesion of the liquid droplet onto the hot-spot surface, such that the contact thermal resistance of the heat transfer was reduced considerably. The hot-spot temperature was cooled from an initial temperature of 172 °C down to 100 °C by four droplets with a volume of 30 µL within 8 min. Furthermore, Hale and Bahadur [52] proposed combining the EWOD liquid handling technique with a two-phase heat pipe cooling technique, called electrowetting heat pipe (EHP). The wick section of a traditional heat pipe was replaced by the EWOD-based electrode pattern. Their experimental results predicted that planar water-based EHP could transfer 1.6 kW over a 1 m distance, which has a cross section of 10 cm by 4 cm with a thermal resistance of 0.01 K/W [53]. On the other side, Chakraborty et al. [54] proposed the enhancement of the cooling performance of an EWOD-actuated droplet by introducing periodic oscillation to the droplet (shown in Figure 5b). A periodic DC voltage signal was applied to the microdroplet, which was varied from 150 to 300 V in terms of voltage and from 25 to 200 ms in terms of delay time. The enhanced heat transfer was caused by the augmented mixing inside the oscillating droplet. Furthermore, they added silver nanoparticles into an oscillating droplet and found that the nanoparticles could enhance both the wettability of the nanofluid droplet and the heat removal capability at the same time [55]. The oscillating nanofluid droplet was actuated by a low-frequency AC electric field (5–500 Hz), which induced the surface deformation and the generation of a droplet surface wave (shown in Figure 5c). The convective heat transfer was enhanced by the surface wave and induced additional evaporative cooling of the nanofluid droplet. Consequently, the overall cooling rate was significantly augmented. In order to increase the heat transfer area for droplet evaporation, Nampoothiri et al. [56] implemented liquid dielectrophoresis by applying a non-uniform AC electric field between the sessile DI water droplet and the electrodes, which, based on their experimental results, could increase the spreading area of the sessile water droplet.

With the aforementioned attractive cooling performances achieved by the EWOD-based methods, it is worth mentioning that all of these methods face serious failure issues of EWOD-based droplet actuation, such as dielectric breakdown and charging [28]. In EWOD, the interface curvature becomes extremely small in the vicinity of the three-phase contact line. As a result, the electric field diverges and the strength of the electric field turns to exceed the dielectric breakdown strength of the thin dielectric layer between droplet and electrode [36,57]. As a result, the whole cooling system is destroyed due to the dielectric breakdown of the insulating layer. Another issue is the coalescence of droplets over time for EWOD devices, which is caused by the contact angle hysteresis and ion adsorption on the solid surface [34]. The contact angle hysteresis results in different contact angle variation with the same applied voltage. The shape variation and moving speed of each droplet becomes different over time. Ion adsorption at the liquid–solid interface is another possible reason to cause droplet coalescence. These two effects also lead to the hysteresis or irreversibility of electrowetting. Improvements can be achieved by using a system of two immiscible liquids and topographically patterned surfaces [34]. However, up to now, little attention has been paid to the reliability of the proposed EWOD-based thermal management systems, which is crucial for the long-term performance of practical applications in the electronics industry. One should also notice the electrothermal effect during EWOD droplet actuation with applied AC electric fields. Joule heating can be induced by the AC electric field in the bulk liquid droplet, and the temperature of the liquid can be increased by several degrees Celsius [58,59]. The electrothermal effect can be solved by increasing the frequency of the applied AC voltage [60]. This heat generated by EWOD is important for lab-on-a-chip applications dealing with biological samples, which are very sensitive to temperature. Nonetheless, the electrothermal effect is compared to the large amount of waste heat from high-performance electronic devices.

## 4. Passive Droplet-Based Microfluidic Methods

**Figure 6:**
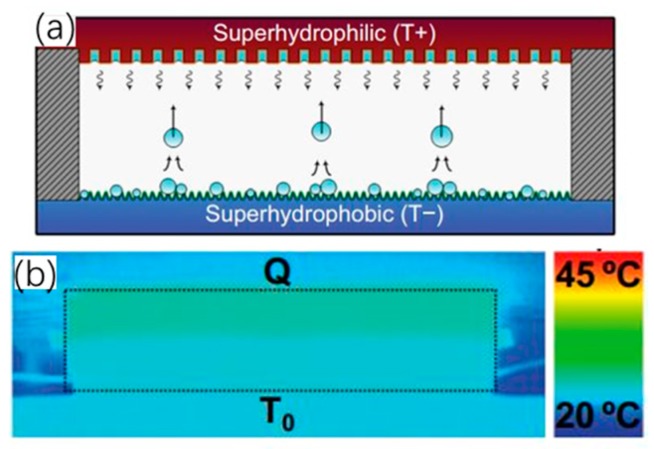
Schematic of the planar phase-change jumping-drop thermal diode. (**a**) Forward mode with self-propelled jumping droplets from the superhydrophobic condenser to the superhydrophilic evaporator, and (**b**) infrared imaging of the diode at steady-state in forward mode. Reproduced with permission from [61]; published by AIP publishing, 2011.

**Figure 7:**
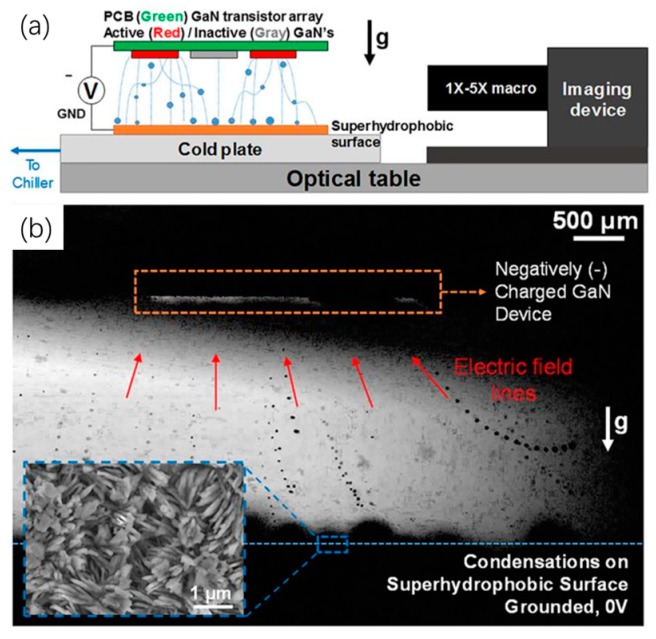
(**a**) Schematic of the experimental setup for jumping droplet cooling of GaN transistors. (**b**) Composite high-speed image of jumping droplet condensation. Reproduced with permission from [68]; published by AIP publishing, 2017.

Apart from the active electrocapillary-based droplet-based techniques, a passive droplet-based liquid handling technology has recently been suggested for electronics cooling. It is called the planar jumping-droplet thermal diode, which was proposed by Chen’s group from Duke University [61]. It consists of parallel superhydrophilic and superhydrophobic plates as an evaporator and condenser, respectively (shown in Figure 6). This new type of thermal diode utilized phase-change heat transfer and the self-propelled jumping droplet against gravity on superhydrophobic surfaces, which was discovered by their group earlier in 2009 [62,63,64]. Unlike the previous thermal diode (e.g., thermosiphon and asymmetric heat pipe) relying on gravity and wick structure, their proposed thermal diode is able to automatically transport liquid condensate back to the evaporator by using the released excess surface energy during the coalescence process of the liquid droplet condensate on the superhydrophobic surface. As such, the droplets can continuously absorb waste heat from the evaporator attached to the hot surface, dissipate the heat out via the condenser, and again return to the evaporator for another cooling cycle. Meanwhile, the thermal rectification is enabled by the thermal condition requirement of the jumping droplet condensate, which needs the superhydrophobic surface temperature to be lower than the superhydrophilic surface temperature to trigger the jumping scheme. The temperature distribution in the thermal diode becomes uniform in forward mode with the jumping droplets, while the heat transfer is blocked with a nonuniform temperature distribution in reverse mode. The jumping droplets tend to repel each other during the jumping flight, which is due to the fact that the droplets gain net positive electrical charges caused by charge separation of the electric double layer at the superhydrophobic surface [65]. To further enhance the heat transfer performance of the jumping-droplet thermal diode, considerable efforts have been made in the fabrication of an advanced superhydrophobic surface [66] and in the electric-field-enhanced (EFE) momentum of jumping droplets against vapor flow toward condenser surfaces (shown in Figure 7) [67,68]. The cooling capacity of EFE jumping-droplet methods can be significantly improved, but an external electrical field needs to be added to the thermal diode system.

## 5. Conclusions and Outlook

With the rapid increase in packaging density and continuous reduction in device size, cooling issues have become a vital problem for extending the progression of Moore’s law and the further development of high-performance electronic devices. High heat flux, non-uniform temperature distribution, and space constraints in compact packaging are three major challenges that advanced thermal management methods must conquer. In terms of emerging cooling techniques, droplet-based microfluidic thermal management methods were first proposed in 2003 and have recently been vigorously investigated. In this review, we focus on droplet-based microfluidic cooling techniques based on surface-tension modulation, especially using the electrocapillary principle. Compared with other macroscale droplet-based cooling methods such as spray cooling, the surface-tension modulation-related microfluidic methods have the advantage of low energy consumption, easy integration with compact size, and selective cooling for hot spots. Among these droplet-based techniques, electrocapillary-based cooling techniques have been suggested as one of the most promising integrated/embedded thermal management approaches, due to their fast liquid actuation response under a low-power electrical field and the precise control of droplet motion with digital manipulation. Three types of electrocapillary-based active cooling techniques have been reviewed: continuous electrowetting (CEW), electrowetting (EW), and electrowetting on dielectric (EWOD).

A passive cooling method was also introduced: the planar jumping-droplet thermal diode, whereby droplet actuation (i.e., jumping) does not require external energy input but uses the released excess surface energy during the coalescence process of the liquid droplet condensate on the superhydrophobic surface. Based on all analyses, droplet-based microfluidic thermal management methods have demonstrated an attractive potential for high performance electronic devices. Lastly, several future research directions are recommended: (1) The development of liquid coolant materials with good thermal property and controllable wettability under electrical field is highly encouraged. (2) Intelligent route design and feedback control for droplet transport based on temperature sensing for hot spots are key to improving system effectiveness. (3) More attention should be paid to the reliability of EWOD-based cooling devices, and further investigations on the mechanisms of dielectric breakdown and droplet coalescence at elevated temperatures are highly recommended for the long-term performance of EWOD-based cooling devices. (4) Investigations on the interference between the electric driving signal for electrocapillary-based cooling system and the digital information signal of the electronic devices are required since the cooling unit is required to be integrated into 3D stacked chips. Further reduction of the driving voltage of EWOD-based cooling techniques is also suggested.

## Figures and Tables

**Table 1:** Thermal management methods for electronic devices and systems.

Category	Cooling Method	Coolant	Removal Heat Flux (W/cm^2^)	Ref.
Passive	Free convection	Air	15	[11]
Active	Forced convection (Heat sink with a fan)	Air	35	[11]
Passive	Heat pipe	Water	250	[12]
Active	Microchannel	Water	1430	[13]
Active	Peltier cooler	Electron	125	[14]
Passive	Pool boiling	FC-72	140	[15]
Active	Flow boiling	HFE 7100	700	[16]
Active	Spray cooling	Water	1200	[17]
Active	Jet impingement	GaIn	2000	[9]
Active	Electrocapillarity	Water	27	[18]

**Table 2:** Physical properties of some liquid metals and alloys. Note: (a) 25 °C; (b) 200 °C; (c) 160 °C; (d) 100 °C; (n) 50 °C; (m) at melting point. Reproduced with permission from [33]; published by Springer, 2007.

Liquid Metal or Alloy	Density kg/m^3^	Melting Point °C	Boiling Point °C	Evaporation Pressure/mmHg	Thermal Cond. W/m·K	Specific Heat J/(kg∙°C)	Viscosity kg/(m·s)	Surface Tension N/m	Ref.
Hg	13546	−39	356.65	1.68 × 10^−3 (a)^	8.4	140	0.15 × 10^−3^	0.47	[33]
Ga	6093	29.98	1983	10^−12^	33.49	343.32	1.89 × 10^−3^	0.735	
Cesium	1796 ^(d)^	28.65	2023.84	10^−6 (d)^	17.4 ^(d)^	236 ^(d)^	—	0.248 ^(d)^	
Rubidium	1470 ^(m)^	38.85	685.73	6 × 10^−6^	29.3 ^(m)^	363 ^(m)^	—	0.081	
Potassium	664 ^(m)^	63.2	756.5	6 × 10^−7^	54.0 ^m)^	780 ^(m)^	—	0.103 ^(d)^	
Sodium	926.9 ^(d)^	97.83	881.4	10^−10^	86.9 ^(c)^	1380 ^(d)^	—	0.194 ^(d)^	
Indium	7030 ^(c)^	156.8	2023.8	<10^−10^	36.4 ^(c)^	270 ^(m)^	—	0.55 ^(m)^	
Lithium	515 ^(b)^	186	1342.3	10^−10^	41.3 ^(b)^	4389 ^(b)^	—	0.405 ^(b)^	
Tin	6940 ^(c)^	232	2622.8	<10^−10^	15.08 ^(b)^	257	—	0.531 ^(m)^	
Water	1000	0	100	—	0.6	4184	0.86 × 10^−3^	0.0717	
NaK	860	−12.6	785	—	0.232 at °C	949	0.522 × 10^−3^	0.12	[31]
Bi-alloys	~9500	47–271	8.4	—	15	197	—	—	
Indalloy 46L	6499	7.6	—	—	—	—	—	—	
Indalloy 51	6499	10.7	—	—	—	—	—	—	
Indalloy 60	6350	15.7	—	—	—	—	—	—	
Indalloy 77	6145	25	—	—	—	—	—	—	
